# 

**Published:** 1929

**Authors:** 


### The Medical Library of the University of Bristol,

WITH WHICH IS INCORPORATED

### The Library of the Bristol Medico-Chirurgical Society.

---

*The following donations have been received since the publication of the list in April,* 1929.

July, 1929.

| | |
|---|---|
| University of Aberdeen (1) .. .. .. | 1 volume |
| Dr. W. S. Bainbridge (2) .. .. .. | 1 ,, |
| Dental Board of the United Kingdom (3) .. | 1 ,, |
| Dr. A. L. Flemming (4) .. .. .. .. | 1 ,, |
| Professor E. W. Hey Groves (5) .. .. | 1 ,, |
| Henry Phipps Institute (6) .. .. .. | 1 ,, |
| Government of India (7) .. .. .. .. | 1 ,, |
| Dr. G. H. Jackson (8) .. .. .. .. | 1 ,, |
| Dr. R. C. Leonard (9) .. .. .. .. | 29 volumes |
| Monsieur A. Lumière (10) .. .. .. | 1 volume |
| Michell Clarke Memorial Fund (11) .. .. | 3 volumes |
| National Institute for Medical Research (12).. | 1 pamphlet |
| Philadelphia General Hospital (13) .. .. | 1 volume |
| Rockefeller Foundation (14) .. .. .. | 1 ,, |
| Surgeon-General of the U.S. Army (15) .. | 1 ,, |

Unbound periodicals have also been received from Professor Hey Groves, Dr. Carey Coombs and Dr. C. Corfield.

### THE ONE HUNDRED AND TWENTY-EIGHTH LIST OF BOOKS.

The figures in round brackets refer to the figures after the names of the donors. The books to which no such figures are attached have either been bought from the Library Fund or received through the *Journal.*

| | | | |
|---|---|---|---|
| **Alport, C. A.** .. .. | *On Nephritis* .. .. .. .. .. .. .. .. .. .. .. | | 1929 |
| **Angle, E. H.** .. .. | *Malocclusion of the Teeth* .. .. | 7th Ed. (11) | 1907 |
| **Barton, E. A.** .. .. | *Essentials of Infant Feeding* .. .. .. .. | 2nd Ed. | 1929 |
| **Bennett, T. I.** .. .. | *Nephritis : its Problems and Treatment* .. .. | | 1929 |
| **Bourne, G. and Stone, K.** | *Principles of Clinical Pathology in Practice* | | 1929 |

| | | | |
|---|---|---|---|
| **Carrington, R. E.** .. | *Notes on Pathology* .. .. .. .. .. .. .. | (9) | 1892 |
| **Chagas, C.** .. .. .. | *On the Practice of Terminal Disinfection* .. .. | | 1928 |
| **Giles, A. E.** .. .. | *Gynæcological Diagnosis* .. .. .. .. .. .. | (9) | 1906 |
| **Gladstone, H. B.** .. | *Infant Feeding and Nutrition* .. .. .. .. | | 1928 |
| **Hamer, Sir W.** .. .. | *Epidemiology, Old and New* .. .. .. .. .. .. | | 1928 |
| **Harrower, H. R.** .. | *Practical Organotherapy* .. .. .. 4th Ed. | (9) | 1922 |
| **Hertzler, A. E.** .. .. | *Technic of Local Anæsthesia* .. .. 4th Ed. | (4) | 1928 |
| **Hess, J. H.** .. .. | *Feeding and the Nutritional Disorders of Infancy* .. .. .. .. .. .. .. 6th Ed. | (11) | 1928 |
| **Hopewell-Ash, E.** .. | *Therapy of Personal Influence* .. .. .. .. | | 1929 |
| **Jones, D. W. C.** .. | *Elementary Medicine in terms of Physiology* .. | | 1929 |
| **Lewis, T.** .. .. .. .. | *Blood Vessels of the Human Skin* .. .. | (11) | 1927 |
| **Leys, D.** .. .. .. .. | *Chronic Pulmonary Catarrh* .. .. .. .. .. .. | | 1927 |
| *Local Analgesia in Dental Practice* .. .. .. .. .. .. .. .. .. | | (3) | 1929 |
| **McCurrich, H. J.** .. | *Treatment of the Sick Poor of this Country* .. | | 1929 |
| **MacWilliam, J. A., and others.** | *Physiological Studies* .. 2nd Series | (1) | 1926 |
| *Practical Methods of Urine Analysis* .. .. .. .. .. .. .. .. | | (9) | 1899 |
| **Saalfeld, E.** .. .. | *Lectures on Cosmetic Treatment* .. .. .. | (9) | 1911 |
| **Sauerbruch, F.** .. .. | *Chirurgie der Brustorgane I*, i. .. .. .. | (5) | 1928 |
| **Smith, F. J.** .. .. | *Forensic Medicine and Toxicology* .. .. 3rd Ed. | | 1929 |
| **Whittaker, C. R.** .. | *Manual of Surgical Anatomy* .. 2nd Ed. | (9) | 1914 |

TRANSACTIONS, REPORTS, JOURNALS, ETC.

| | | |
|---|---|---|
| Methods and Problems of Medical Education. 12th Series .. .. | (14) | 1929 |
| Progressive Medicine. June, 1929 .. .. .. .. .. .. .. .. .. .. .. | | 1929 |
| Report of the Henry Phipps Institute .. .. .. .. .. .. .. .. .. | (6) | 1928 |
| Report of the International Congress on Military Medicine and Pharmacy. 1927 .. .. .. .. .. .. .. .. .. .. .. .. .. .. .. .. .. .. | (2) | 1929 |
| Studies from the Laboratories of the Philadelphia General Hospital. v. | (13) | 1928 |
| Surgical Clinics of North America. Feb., 1929 .. .. .. .. .. .. .. | | 1929 |
| Transactions of the 7th Congress of the Far Eastern Association of Tropical Medicine. Vol. I. .. .. .. .. .. .. .. .. .. .. .. .. .. .. .. | (7) | 1928 |

### Local Medical Notes.

EXAMINATION RESULTS.

F.R.C.S.—Robert Victor Cooke, M.B., Ch.B., Bristol.

**University of Bristol.**—Students of the University have recently passed the following examinations :—

M.B., CH.B.—*First Examination. Pass:* Dorothy E. Barber, A. G. W. Branch, Rosalind M. S. Derham, Violet Fry,

A. V. House, A. D. Jones, C. H. G. Price, C. F. Rivington, S. Roberts, Mary G. Thomas, Marion L. Tryon.

M.B., CH.B.—*Final Examination* (*Part I.*). *Pass: In Forensic Medicine and Toxicology only*, A. J. B. Miall, H. M. Strover, W. L. Sleight.

M.B., CH.B.—*Second Examination* (*Part II.*). *Pass:* J. R. Gibbs, H. James.

M.B., CH.B.—*Final Examination* (*Part II.*). *Pass:* With Second Class Honours, R. A. S. Cory (with distinction in Pathology, Medicine and Obstetrics), R. J. Krogh (with distinction in Medicine and Obstetrics). *Pass:* Isabella J. Armstrong, A. C. Fisher, April D. James (with distinction in Public Health). *In Group II.* (*Completing Examination*): E. Southam. *In Group I.:* N. D. Gerrish, Mabel F. Potter. *In Group II.:* N. L. Price.

B.D.S.—*Third Examination. Pass: In Dental Metallurgy and Dental Mechanics* (*Completing Examination*), Muriel E. S. Cosh. *In Dental Materia Medica* (*Completing Examination*): W. Forsyth. *In Dental Metallurgy and Dental Mechanics only:* M. J. Ryan.

L.D.S.—*First Professional Examination. Pass: In Physiology and Anatomy* (*Completing Examination*), R. E. W. Smith. *In Physiology and Anatomy only:* Helena C. L. Blinkworth, G. N. Minifie, J. H. G. Smith.

L.D.S.—*Second Professional Examination. Pass: In Dental Materia Medica only*, E. V. O'Hara, H. L. Saunders.

L.D.S.—*Final Professional Examination. Pass:* C. R. H. Edwards, L. W. G. Jones.

CONJOINT BOARD.—M.R.C.S., L.R.C.P. (*Final Examination*), C. E. Brierley (in Surgery).

M.R.C.S., L.R.C.P.—R. J. Krogh, J. V. Lucas, K. H. Pridie, N. C. Tanner, G. J. S. West.

L.D.S., R.C.S.—*First Professional Examination* (*Part I.*). *Pass:* E. R. Dowland, T. A. A. Eaves, D. R. Jones, W. R. R. Mewton. *Part II. Pass:* G. H. Dakers, E. S. Edwards, W. U. Harwood, A. M. Watson, J. H. White. *Part III.* (*b*). *Pass:* E. R. Dowland, I. M. Ayliffe, W. A. Duly, R. E. Morgan.

